# I Didn’t Want the Psychotic Thing to Get Out to Anyone at All: Adolescents with Early Onset Psychosis Managing Stigma

**DOI:** 10.1007/s11013-024-09859-3

**Published:** 2024-06-13

**Authors:** Dea Gowers Klauber, Sofie Heidenheim Christensen, Anders Fink-Jensen, Anne Katrine Pagsberg

**Affiliations:** 1 Child and Adolescent Mental Health Center, Copenhagen University Hospital – Mental Health Services CPH, Copenhagen, Denmark; 2 Department of Clinical Medicine, Faculty of Health and Medical Sciences, University of Copenhagen, Blegdamsvej 3B, 2200 Copenhagen, Denmark; 3 Laboratory of Neuropsychiatry, Department of Neuroscience and Pharmacology, University of Copenhagen, Nordre Ringvej 26-67, 2600 Glostrup, Denmark; 4 Psychiatric Center Copenhagen, Mental Health Services, Capital Region of Denmark, Copenhagen, Denmark

**Keywords:** Psychosis, Adolescents, Stigma, Phenomenological, Denmark

## Abstract

The impact of stigmatisation on adults with mental illnesses has been thoroughly demonstrated. However, little is known about experiences of stigmatisation among adolescents with mental illness. Through semi-structured interviews with 34 Danish adolescents (14–19 years) diagnosed with psychosis, this study explores adolescents’ experiences of psychosis stigma. On the basis of phenomenological analysis, we find that stigmatisation is widely experienced, and psychosis is generally regarded as more stigmatising than co-morbid mental illnesses. The participants engage in different strategies to manage possible stigma, especially strategies of (non-)disclosure. Disclosure is experienced as both therapeutic and normative, but also bears the risk of stigmatisation, and is therefore associated with numerous considerations. Being understood when disclosing is central to the participants, and lack of understanding from others is a continuous challenge. Nevertheless, participants experience benefits when feeling understood by people they confide in and can to a degree create the grounds for this through centralising aspects of their experiences of psychosis and mental illness. We argue that disclosure is both a stigma management strategy and a normative imperative, and that being understood or not is a challenge transcending stigma definitions.

Clinical trial registration: Danish Health and Medicines Authority: 2612-4168. The Ethics Committee of Capital Region: H-3-2009-123. ClinicalTrials.gov: NCT01119014. Danish Data Protection Agency: 2009-41-3991.

## Introduction

Erving Goffman defined stigma as “an attribute that is deeply discrediting […] turning a whole and usual person to a tainted and discounted one” (Goffman, 1974), and distinguished between discredited (non-concealable) attributes, e.g. gender, and discreditable (predominantly concealable) attributes, e.g. mental illness. Goffman’s work emphasises stigma as a social process, playing out in the context of social relationships and shaped by cultural structures. The impact of the discreditable stigma of mental illness on adults has been thoroughly investigated, showing stigma leading to self-stigma with feelings of shame and lowered self-esteem, non-acceptance and non-adherence to treatment, social withdrawal and diminished quality of life (Markowitz, 1998).

In contrast, few studies have examined experiences of stigmatisation in youth with mental illness (Ferrie et al., 2020) and none in children and adolescents with early onset psychosis (onset before the age of 18 years, which affects 0.05–0.5% of the population (Gurholt et al., 2022)). This lack of insight is problematic for two reasons: (1) Psychotic illnesses are particularly stigmatised, compared with other mental illnesses, with misconceptions of people with psychosis being violent or less intelligent (Link et al., 1999; Schulze & Angermeyer, 2003; Wright et al., 2011), a problem that holds true across five European nations and the Nordic countries (Hellström et al., 2023; Pescosolido et al., 2008) and that may be increasing despite de-stigmatisation efforts (Pescosolido et al., 2010; Schomerus et al., 2022). (2) Stigmatisation experiences of children and adolescents are underexamined, yet may be qualitatively different to adults (DeLuca, 2020; Heary et al., 2017), with adolescents being potentially extra vulnerable to the negative effects of stigma, due to a greater need to “fit in” with peers (Ferrie et al., 2020), which can potentially enhance the perceived risks of peer rejection. The high risk of stigmatisation and detrimental effects thereof make it of great importance to understand how stigmatisation of psychosis affects children and adolescents.

We explore young people’s experiences of stigmatisation of psychosis and their strategies for managing the threat of stigma. Thereby the study offers rare insight into the lives of a group of adolescents who are at increased risk of stigmatisation and whose stigma experiences may differ from adults.

## Methods

The study is part of the Tolerability and Efficacy of Antipsychotics (TEA) trial, an investigator-initiated, randomised, double-blinded, multicentre trial comparing the beneficial and harmful effects of two antipsychotics (aripiprazole and quetiapine) in 113 children and adolescents with first-onset psychosis. Children and adolescents of both sexes aged 12–17 years with non-organic and non-drug-induced psychosis were included. In the following, only the design and methods of the present qualitative study are outlined. For further details on inclusion and exclusion criteria and the full TEA trial, see the published study protocol (Pagsberg et al., 2014) and study results (Pagsberg et al., 2017).

### Procedures

The study is based on semi-structured interviews with 34 participants from the TEA trial. The interviews took place from December 2012–April 2015. All participants had been enrolled in the TEA trial and randomised to a 12-week treatment. One year after inclusion, participants were reassessed, and based on the aim of forming a representative group with respect to age and sex, a subsample were invited to participate in a semi-structured interview. The study procedures were explained to participants and their guardians, and then guardians and participants 18 years and above provided written informed consent prior to participation. All participants were assured anonymity. Most of the interviews were carried out in hospital offices, and the rest in the participants’ private rooms in the hospital in-patient ward or at their treatment home. All interviews were conducted by the same interviewer (Klauber) and lasted between 20 and 90 min. Differences in duration were primarily associated with the participants’ ability to keep focus (if they repeatedly lost focus, the interview was shortened). Interviews were audio-recorded and transcribed verbatim. The study and procedures were approved by the National Committee on Health Research Ethics (Protocol No. H-3-2009-123) and the Danish Data Protection Agency (Journal No. 2009-41-3991).

### Participants

**Fig. 1:**
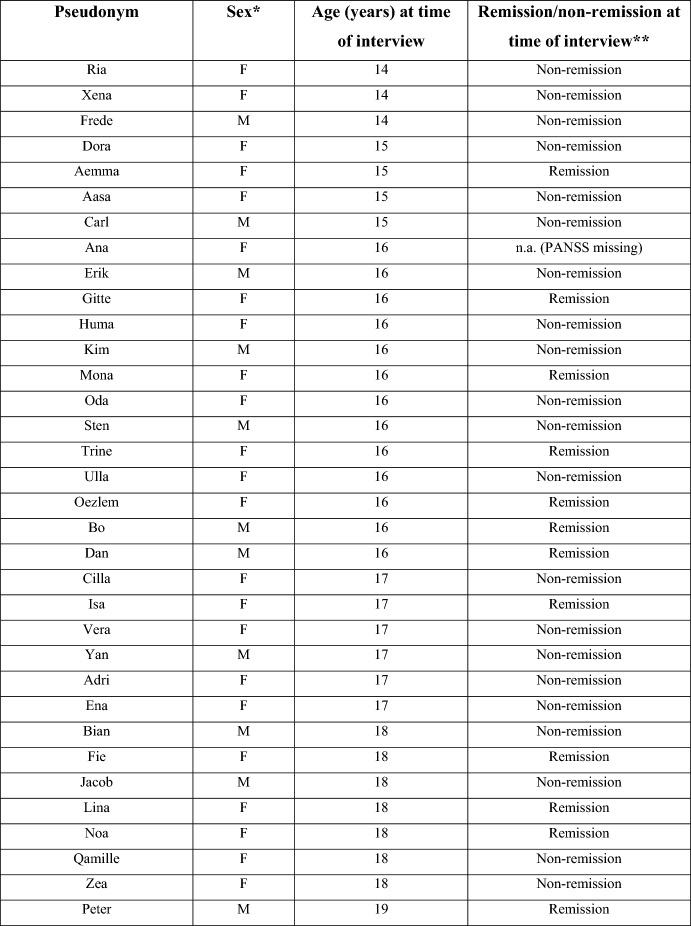
List of interlocutors. *Male or female. **Remission defined as a score of ≤ 3 (severity is mild or less) on eight selected PANSS-items: *P1*: delusions, *P2*: conceptual disorganisation, *P3*: hallucinatory behaviour, *N1*: blunted affect, *N4*: social withdrawal, *N6*: lack of spontaneity, *G5*: mannerisms and *G9*: unusual thought content. *F* female, *M* male, *n.a.* not applicable

In total, 23 girls and 11 boys aged 14–19 years participated in interviews. All had received a diagnosis of psychosis (verified with Kiddie Schedule for Affective Disorders and Schizophrenia – Present and Lifetime (Kaufman et al., 1997)) at baseline. Of the interviewees, 20 were diagnosed with paranoid schizophrenia (F20.0); 9 with schizoaffective disorder, depressive type (F25.1); and 5 with either other psychotic disorder (F28.0), unspecified schizophrenia (F20.9), brief psychotic disorder (F23.9), schizoaffective disorder, bipolar type (F25.0) or undifferentiated schizophrenia (F20.3). Several had psychiatric comorbidities. The adolescents were from Denmark (geographical areas: Zealand, Funen, Southern Jutland and Bornholm). None of the adolescents lived by themselves; some lived at home with parents or in a foster family, while others lived in residential treatment homes. Some of the adolescents attended ordinary primary school or high school, whereas others attended school for youths with special needs. Of the 34 adolescents, 12 (35.3%) were in remission at the 1-year follow-up (Fig. 1).

Hereafter, the children and adolescents are termed interlocutors. This choice reflects the analytically grounding understanding that they are not equal participants in the study, determining the focus and methodology, nor are they passive informants but rather interlocutors who, in conversation with the interviewer, guide and mediate the study.

### Methodological Approach

The overarching research question of the study was how adolescents experience stigma with regards to psychosis. A qualitative and phenomenological methodology was chosen to explore the perspectives and lived experiences of stigmatisation of the adolescents. The interviews were semi-structured with open-ended questions inspired by the Teen Subjective Experience of Medication Interview (TeenSEMI; Jenkins et al., 2005) adapted from the adult version and Experiences with the Stigma of Mental Illness Questionnaire (Stuart, 2005). We improved the interview guide through a pilot study with two patients in medical treatment for psychosis who were asked to evaluate the interview. On the basis of the feedback, more open-ended questions were included, and the interviews were adjusted to let interlocutors guide the interviews more.

The interviews focused on daily life after diagnosis, with special focus on relationships with family, peers and teachers. The interviewer did not use terms such as ‘stigma’ or ‘discrimination’, so as not to lead the interviews (the term ‘stigma’ was not mentioned at all, and ‘discrimination’ only once by an interlocutor). While some interlocutors shared detailed descriptions of reactions to disclosure, others elaborated less on their experiences, only providing very short answers, and pausing several times during the interviews. In these instances, the interviewer attempted to avoid leading interlocutors, instead using prompts to elucidate further descriptions.

As such, the experiences recounted by interlocutors are results of an interview process mediated by the interviewer, and further mediated by the analytical process. We therefore consider the perspectives and experiences in the study as emergent through a joint process, with interlocutors, interviewer and analyst as active participants in the knowledge produced (Rapley, 2004). The in-depth solo interviews allowed for the interviewer to delve into both the history and everyday life of the interlocutors, and gave them time to explore general themes with lived examples.

By approaching the study phenomenologically and emphasising the mediated nature of the analysis, we started with the experiences of the interlocutors, rather than a pre-formed hypothesis or theory. This is especially important in studies of stigmatised groups performed by researchers who are not part of the group under study; see, for instance, Schneidre (1988) on the critique of non-stigmatised experts prioritising social theories over experiences and perceptions of the people they study.

### Analytical Process

All interviews were read in their entirety to identify recurring themes. The interviews were coded in NVivo© to ensure effective data handling (Dhakal, 2022). Due to a career change by the interviewer, the analysis was carried out by an analyst (Christensen), who had not been part of the interview procedures. This added the challenge of analysing “at a remove” from the data and the contexts in which they were generated (Irwin, 2013). Analysis being conducted by a researcher other than the interviewer also meant that a certain amount of detachment was possible (Szabo & Strang, 1997), as analysis was carried out without prior knowledge of specific presuppositions guiding the interviewer. Discussions between interviewer and analyst secured that contextualising knowledge was not lost.

Analysis was conducted as continuous comparison with stigma as a sensitising concept. The analyst started with in vivo coding, and then inductively built a coding matrix, gathering emic codes into overarching themes. Coding was handled in three stages: continuous coding, analysis and re-coding. First, approximately a third of the interviews were coded, moving from meaningful units to emic codes, then overarching themes, and tentative analysis. Second, another third of the interviews were coded, providing new codes and refining existing ones, leading to the re-coding of the first set of interviews and further development of themes and analysis. Finally, the remaining interviews were coded separately, and compared with the analysis developed in stages 1 and 2 – both confirming the developed analysis and nuancing it. Subsequent re-coding of the first two-thirds of interviews was limited, as codes had stabilised by this point.

The analysis was advanced using theoretical frameworks and concepts developed by Erving Goffman (1974), Nancy Herman (1993), Chaudoir and Fisher (2010), Roy Cain (1991), and Molly Leszcz and Irvin Yalom (2005).

## Analysis

Stigmatising experiences are part of nearly all the young interlocutors’ experiences, and they are affected by both experienced and anticipated stigma (Earnshaw & Chaudoir, 2009; Vogel & Wade, 2022). Experiences of stigmatisation include friends and family withdrawing from the relationship, being treated differently (e.g. like a fragile person), being labelled, being reduced to the psychotic illness, being bullied, and experiencing the loss of a job opportunity. How these experiences are regarded differs; for instance, being treated differently because of the illness was by some described as stigmatising, while others appreciated considerations of their special needs:

My dad does [treat me differently] […] he pays more attention to me than my younger brother… just because of the diagnosis [schizophrenia]… and feels that it’s much more important to help me. (Ria, 14 years)

I had a really good boss […] he could see, when it started […] ‘you can just grab your stuff and head on home’ […] he understood, and he was considerate of me. (Lina, 18 years)

### (Non-)disclosure: Strategic Management of Stigma

When I meet people, I have to find out who they are, and I have to consider whether they will even be able to handle it […] Because there are some who, if I told right away [about the illness], would just withdraw. And those who I feel can handle it, of course I tell them about it, and usually I also tell it, when they have seen me be well-functioning. (Jacob, 18 years)

Throughout the interviews, it is apparent that the interlocutors are not resigned to the stigmatisation, but rather constantly engage in stigma management (Goffman, 1974), looking for ways to navigate the field of possible and actualised stigma. Jacob did not simply accept the risk of withdrawal; rather, he utilised two strategies: (1) making sure people see him ‘function’ before disclosing his illness, and (2) timing, adjusted to his predictions of recipients’ reactions (whether they will be able to handle it, or whether they will withdraw). Whitley and Campbell, who similarly find their interlocutors engaged in stigma management, describe psychological (discourse that ‘normalises’ or ‘universalises’ mental illness) and behavioural (efforts to ‘blend in’) strategies (Whitley & Campbell, 2014). In contrast, interlocutors in our study primarily employ a variety of narrative strategies. Much as with Dinos et al., who found that, in their interviews with adults with mental illness, “Concern about disclosure emerged as a major theme” (Dinos et al., 2004), strategic management of stigma among the interlocutors of our study centres around disclosure.

Nancy Herman, in her study of ex-psychiatric patients, outlines four strategies of information control: (1) selective concealment, defined as “selective withholding or disclosure of information about the self perceived as discreditable” (Herman, 1993); (2) therapeutic disclosure, or disclosure to trusted others to renegotiate personal perceptions of the stigma; (3) preventive disclosure, or disclosure in an effort to avoid anticipated problems or get ahead of possible rejection; and (4) political activism, defined as a collective management strategy through participating in activist groups (Herman, 1993). These strategies closely resemble, but also differ from, the narrative stigma management apparent in the narratives of the interlocutors of our study. The sections below will highlight similarities and differences in management strategies.

### Disclosure of Psychosis

There was no one who knew that it [being hospitalised] was because of me being psychotic. They [my classmates] just thought it was because of my depression and anxiety […] because I didn’t want the psychotic thing to get out to anyone at all. (Mona, 16 years)

Carefully choosing disclosure of only certain aspects of experiences with mental illness was one of the foremost strategies used to manage the risk of stigma among the interlocutors. While a few interlocutors found disclosure of for instance depression to be “less personal” than disclosure of psychosis, several interlocutors were very concerned with not disclosing, in particular, the psychotic aspect of their experience. Isa said that she was “very happy” that she was diagnosed with depression with psychotic symptoms:

It makes it a bit easier, when I tell people what I’ve been through, that it’s depression and not psychosis. Because it’s easier for people to understand that you get over depression, than that you get over psychosis. (Isa, 17 years)

The strategy of selecting aspects when disclosing resembles the selective concealment strategy (Herman, 1993). However, selective concealment does not delve into the distinctions between discreditable information that are made by interlocutors of our study. While mental illnesses are treated by interlocutors as discreditable, disclosure of psychosis is experienced as particularly stigmatising, and is as such subject to more intense concealment. The reasons for this according to the interlocutors differ: fear of social exclusion if psychosis is disclosed; the experience that, once disclosed, psychosis became the focus; not matching the interlocutor’s own nuanced experience of their challenges; and a public perception that you cannot “get over” psychosis (and thus always being perceived as ill). The last reason matches research on the role of neurobiology in stigma reduction efforts; Phelan finds that genetic attribution of mental illness is often associated with higher levels of stigma, which she associates precisely to the perception of permanence (Pescosolido, 2013; Phelan, 2005). The choice of the selective concealment strategy, with distinctions between discreditable information, was thus both influenced by and revealed the interlocutors’ perception of psychosis as especially stigmatising compared with co-morbid mental illness. It is important to note that, though the interlocutors differentiated psychosis from their co-morbid mental illnesses, when they spoke of psychosis as especially stigmatising, they primarily spoke about their mental illnesses and disclosure without distinguishing between their different diagnoses.

### A Complex of (Non-)disclosure Considerations

When speaking about their choices of narrative stigma management strategies, the interlocutors described a whole host of situational and interwoven considerations centred around (non-)disclosure. These considerations concerned what to disclose (ranging from openness about both symptoms and diagnosis to vague descriptions of “having a hard time”), to whom (considering, e.g., the level of social maturity and the kind of experiences the recipient had), when (e.g. early in the relationship, or after having established themselves as “well-functioning”), and how (verbally or through cues such as taking medicine). Below is an example by Qamille, who selectively tailored what aspects she disclosed depending on the closeness of the relationship:

My mom, she’s the one I like use [to talk to about symptoms and difficulties]. […] The rest [of the family] just know I’m having a hard time […] and maybe they know a bit about the depression, but I’ve said that I don’t want them to know too much […] because I don’t really have like a close relationship with them. (Qamille, 18 years)

**Fig. 2:**
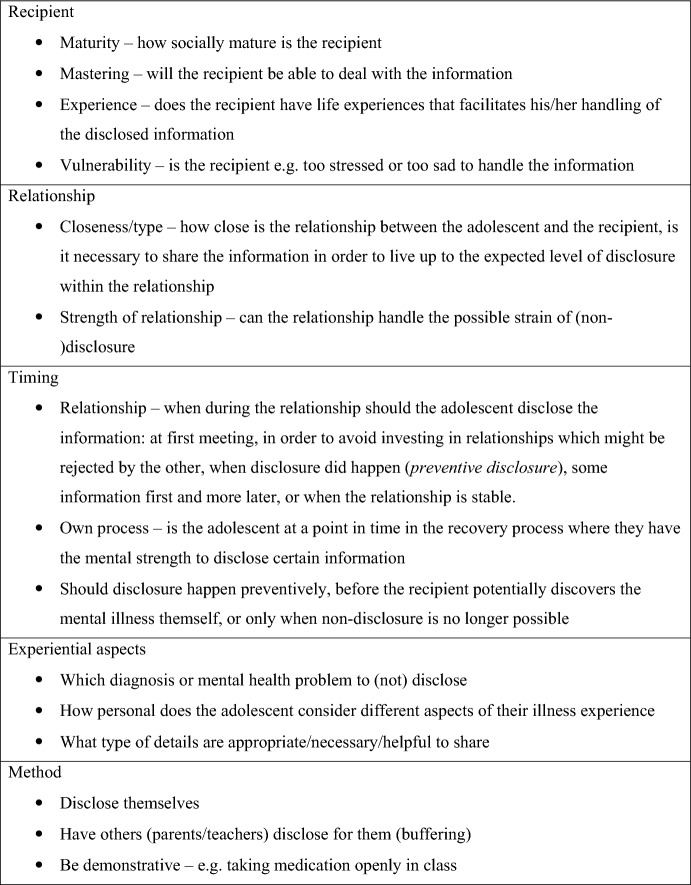
Domains of interlocutors’ considerations concerning disclosure of mental illness

Their considerations form a complex matrix the adolescents draw upon when managing the risk of stigmatisation through (non-)disclosure strategies. Fig. 2 shows (some of) the manifold considerations the interlocutors have when navigating this field of both possible stigma and its impacts on themselves, their relationships and their disclosure recipients. We divide these considerations into five domains: recipient, relationship, timing, experiential aspects and method. Jacob’s considerations incorporate two of these (recipient and timing), while others described disclosure as an act necessitating a certain amount of strength – something you need to get ready for:

I’m trying to, like, to make myself, stronger to do it. To tell. (Oda, 16 years)

Even though the interlocutors’ (non-)disclosure considerations and strategies are recounted as ways to navigate the field of possible stigma, an additional recurring concern was to ensure that friends and family were not hurt or worried.

I don’t know if she [mother] knows [that I still have feelings of derealisation], but I just prefer not to tell her… because I feel bad for her. I don’t want her to worry about me […] and she’s also very stressed. (Ulla, 16 years)

### (Non-)disclosure: A Trial-and-Error Approach

The considerations outlined in the matrix in Fig. 2 and employment of the abovementioned strategies is a way for the interlocutors to manage the risk of stigma. However, choosing strategies becomes something of a trial-and-error approach due to the complexity of the situation in which the outcome of (non-)disclosure hinges on several interconnected factors. Almost all participants have had experiences with strategies not yielding the hoped-for outcome.

I had told her [classmate] some of the things I’ve tried to do [suicide attempt and self-harm], because we were really, really good friends, and she went around and told people […] then suddenly they shouted in class ‘Oh no, I’m seeing something that wants to kill me’ […] if I have told people, they have used my illness against me. […] I’ve become afraid of what I tell people. And many feel that I’m not honest enough with them about these things so… They can tell something is wrong. Many get a bit annoyed or believe that I don’t trust them enough to tell them how I feel, because I don’t tell them what has happened or what it is, why I behave like I do. And then, people start to not truly trust me. (Cilla, 17 years)

After experiencing withdrawal and bullying following her selective disclosure, Cilla revised her strategy. She then employed selective non-disclosure, only to be confronted with a consequential lack of trust. As Goffman has found, close relationships with others are often societally ratified by mutual disclosure of invisible failings (Goffman, 1974). We largely expect at least a degree of personal disclosure from our friends, and similarly feel deceived to find out that close relations have concealed personal information from us. As Cilla experienced, both disclosure and non-disclosure thus may lead to undesired outcomes, e.g., bullying and a lack of trust.

### Stigma Management as an Ongoing Process

Earlier studies have demonstrated that having a discreditable stigma – a stigma not readily apparent to an observer – positions people in a complex situation where they have to navigate between lived experiences of (non-)disclosure, their own learned perceptions of the stigma in question and the particular relationship itself (Goffman, 1974). As Herman likewise notes, the lived experience of what to disclose, to whom, how and when is a crucial part of building experience with what to consider when (not) disclosing (Herman, 1993). This is also apparent in our study, e.g., Jacob’s considerations when meeting new people. His lived experience of disclosure was that some people would be able to handle it, while others would withdraw, and his strategy of making sure the last group see him “function” before disclosing shows both his perception of the stigma of psychosis as “not well-functioning” and his disclosure considerations built on previous experiences.

For the interlocutors it is thus not a simple question of whether to disclose or not. The continuous interplay between the interlocutors’ choices of disclosure strategies, own perceptions of psychosis stigma, a myriad of considerations and experiences of disclosure highlights that stigma management is an ongoing process. This mirrors the Disclosure Process Model (DPM), developed by Chaudoir and Fisher. The DPM consists of five components: antecedent goals (e.g. therapeutic and preventive disclosure), the disclosure event itself, mediating processes, outcomes (e.g. elicitation of social support and greater dyadic intimacy) and a feedback loop wherein future decision-making regarding disclosure is impacted (Chaudoir & Fisher, 2010). The DPM emphasises disclosure as a process where mediating processes and outcomes constitute lived experiences that feed back into disclosure strategies. In our study, the processual aspect is especially highlighted by how gradual disclosure was. Telling peers and family about challenges, symptoms and diagnosis did not happen all at once, and each disclosure event prompted new considerations about disclosure. Disclosure among interlocutors of our study is as such an ongoing process, at times starting with disclosure of being stressed or depressed and gradually including details of psychotic symptoms and diagnosis.

The feedback loop component of the Disclosure Process Model posits each disclosure event as part of the ongoing stigma management process where the outcome of one disclosure event affects the potential of the following one. Negatives outcomes such as stigmatisation may inhibit subsequent disclosure (Chaudoir & Fisher, 2010). However, the DPM does not take into account that outcomes of non-disclosure also create feedback. Cilla’s experiences of stigmatisation impact her disclosure considerations, but so too does the experience of negative outcomes of non-disclosure.

The interlocutors’ own perceptions of stigma, many considerations (including considerations of the relationship in question) and experiences of both disclosure and non-disclosure and the outcomes thereof all feed into their choices of (non-)disclosure strategies. However, the dilemma of possible negative outcomes of both disclosure and non-disclosure facing the interlocutors, as exemplified by Cilla’s account, also highlights the cultural structures influencing the interlocutors’ strategic choices and stigma management processes. Though the analysis underscores the agency of the interlocutors, the structural influences cannot be ignored. One such structure is the normativity of openness.

### Openness: Both Therapeutic Disclosure and Normative Context

Several interlocutors recounted positive outcomes from disclosure, such as gaining support and understanding from others. Several other studies of stigma management have described therapeutic disclosure as a way to enhance self-esteem, solicit support or share emotional burden (Cain, 1991; Herman, 1993). For instance, the interlocutors Sten and Huma:

Sten (16 years): They [teachers] have to know […] I… feel safer, when they know [that I have a mental illness]… because they have to know what to do, if something were to happen. […]

Interviewer: So, do you think that them knowing makes it possible for them to make special considerations for you that…

Sten: Yes… I think so

I can talk more openly about that stuff now because people, like, know that I’m ill. So, I can say ‘I’m afraid now’ or like, ‘I’m uncomfortable now’. (Huma, 16 years)

While there are positive reactions of, e.g., acceptance, unchanged relationships with others and reactions of increased acknowledgement of special needs after disclosure, the interlocutors did not engage in political disclosure strategy, as several interlocutors in the study by Herman did (Herman, 1993).

Huma (16 years): I really support the campaign called One of Us [a Danish project to de-stigmatise mental illness]

Interviewer: You haven’t had anything to do with it yourself or…?

Huma: No… I don’t really dare… I think I’m a bit too young to like say: ‘Hey, listen…’

The reticence of our adolescent participants to engage in political disclosure compared with Herman’s adult participants is especially interesting, as a societal and political push for de-stigmatisation and de-tabooisation of mental illness seems to have given rise to a push for openness and disclosure. This mirrors a trend in how health professionals in the early 1970s began framing disclosure of homosexuality as a central aspect of social and psychological well-being and linking normative disclosure to identity development (Cain, 1991). The reasoning behind both these movements is twofold, having to do both with the effect of therapeutic disclosure and with the idea of widespread disclosure leading to de-stigmatisation in the broader society. Cain explores how the move towards normativisation of disclosure, where being out is increasingly considered the ‘right’ way to be gay, has led to pathologisation of concealed homosexuality. The same pathologisation cannot be said of the move towards openness about mental illness, but by drawing on Cain’s analysis, we can understand why interlocutors recount experiences of therapeutic disclosure, which in turn motivate new disclosure events while simultaneously describing a social pressure to disclose, for instance, with Cilla, as mentioned above, as well as Huma:

They [my teachers] thought I should tell my class. That I, umm, should be more open about it [the mental illness]. […] Then they asked out loud in class ‘You have obsessive thoughts, right?’, and then I said ‘no’ […] then they said they knew that I had. […] They kept pushing me to, like, tell the class. (Huma, 16 years)

Huma’s experience of teachers ‘outing’ her without her consent is an extreme one compared with the other interviews, but when the interlocutors are making choices about what to share with whom, they do so in the context of this normative openness. As one of the youngest interlocutors, Frede, said his mother had advised him “it’s better to be open about it than hiding it”. While disclosure is one strategy to, e.g., solicit support, it is also a daring strategy which can end in stigmatisation; as Cilla said, “my classmates were very immature, they weren’t ready for it”. One reason for the contrast in the utilisation of political disclosure, compared with Herman’s adult participants, might be the adolescents’ young age. As Huma said, “I’m a bit too young”. Other interlocutors similarly commented on the immaturity of their peers, stating that they were not mature enough to be able to react in non-stigmatising ways to disclosure.

The normativity of openness, as exemplified by the experiences of Huma and Frede, and expectations of mutual disclosure for relationship ratification are external factors that influence decisions of disclosure. The DPM focuses heavily on internal motivations and goals, but we argue that they are not sufficient for understanding (non-)disclosure decisions. The importance of these external factors takes on even more weight considering this specific age group. As adolescents are more peer oriented than adults (Ferrie et al., 2020), the need to live up to expectations of disclosure for peer-relationship ratification may be even more motivating for adolescents than for adults. With regards to the normativity of openness, adolescents are structurally positioned under adult authority and care, which creates expectations for compliance and acquiescence that are not similarly expected of adults (Montreuil et al., 2020). That means they may be more vulnerable to pressure from, for instance, well-meaning adults who motivate disclosure. Furthermore, also discouraging this touted openness is a pervasive feeling of not being understood when disclosing.

#### They Don’t Get It: Beyond Stigma

Interviewer: Do you remember what was going on inside you at the time, when you said you didn’t want to tell others?

Peter (19 years): Hmm, I think it was like, that fear that people wouldn’t be able to… understand why, that they like can’t understand that you… feel the way you do.

Though the risk of stigmatisation is part of the interlocutors’ disclosure considerations and strategies, just as powerful is the fear and experience of not being understood. “They don’t really understand” and ”they don’t get it” are some of the most recurring sentences in the interviews, and as a theme it permeates the interlocutors’ stories side by side with, and often undifferentiated from, stigmatisation. The interlocutors use ‘not understanding’ to explain peer and family reactions to disclosure, in frustration, when having been met with “I understand”, as well as a reason for not disclosing:

They [teachers and classmates] listened and expressed that they completely understood what I was going through, but… I did know that they didn’t. (Lina, 18 years)

I don’t feel like I can open up to them [teachers], because many don’t really know what it is like [having a mental illness]. (Cilla, 17 years)

‘Not being understood’ impacts disclosure decisions in three different ways – before, during and after: Before, the fear of not being understood exists pre-disclosure and sometimes discourages disclosure. During, the fear guides choices of selective disclosure, especially only disclosing elements that the interlocutors think will be understood, e.g., having a bad day or being stressed. After, it manifests as frustration and in interpretation of reactions (they react like this because they don’t understand).

If I say to one of them [my well-functioning friends], ‘I feel like this and this’ […] they almost fall off the chair […] ‘Are you sure, you shouldn’t go back to the hospital?’. […] It is so alien to them […] It isn’t something you can see, that’s also why it’s so difficult to explain how you feel about it. It’s a bit like trying to explain how water tastes, and that is really difficult. Especially to someone who has never tasted water. (Fie, 18 years)

What is expressed by the interlocutors is that their experiences are so foreign to others that even disclosure cannot bridge the gap. It is a powerful cause for fear, frustration and distance in the relationships of the interlocutors. These experiences and fears of not being understood spill over the boundaries of the concept of stigma. Not being understood does not entail devaluation and exercise of power – both primary elements of one of the most widely applied definitions of stigma (Andersen et al., 2022; Link & Phelan, 2006) – nor is it part of the seven dimensions of public stigma, which include social distancing, prejudice, exclusion, negative affect, treatment and disclosure carryover, and perceptions of dangerousness (Pescosolido & Martin, 2015). But even though this experiential element is not encapsulated in the stigma definition, the experiences of not being understood are often mentioned in contexts of precisely such experiences of stigmatisation, for instance, Cilla talking about her peers:

Interviewer: How do they [classmates] use it against you?

Cilla (17 years): Like I said earlier [bullying in school], and a lot of them don’t understand. How I feel.

#### Those That Understand: The Malleability of Universality

[My friend] also has these days where she can’t get out the door… [she] has just as galloping ADHD as I do, and also borderline or something […] so she feels a lot like I do, and that’s why she is one of those where I can sit down and actually tell, how it happens in my head. […] it isn’t gibberish to her ears. (Fie, 18 years)

This pervasive feeling of not being understood is contrasted with those that do understand. Most interlocutors had peers, family members or other significant adults they categorised like this. Interlocutors highly valued these relationships, though only few articulated why they are important:

They [children with a psychiatric diagnosis] also have some problems they struggle with, so if you have kind of the same problem, you can talk a bit about it. […] that’s a bit easier than like being with someone who doesn’t really know so much about it and… has never had a crisis themselves. (Frede, 14 years)

In *The Theory and Practice of Group Psychotherapy*, Molly Leszcz and Irvin Yalom identify 11 primary therapeutic factors in group therapy, one of which is universality. Universality is a therapeutic mechanism, conceptualising connectedness to others also suffering, as well as the experience of not being unique in one’s own wretchedness (Leszcz & Yalom, 2005). Huma encapsulated the experiences of universality described by, e.g., Fie and Frede, when she said: “it’s nice, knowing I’m not the only one”.

What stands out in our analysis is the malleability of this universality. Some interlocutors described a need for very closely related experiences, whereas others were able to establish a shared understanding through more parallel experiences. The differing need can be seen in the following two quotes:

Huma (16 years): My best friend, she also has an illness. So, we can compare a bit […]

Interviewer: Is it also a mental illness or…?

Huma: No, it’s morbus crohn

Interviewer: So, it’s a bit different

Huma: Yes, but we can compare, when you have a bad time in your life or such […] also that thing about […] if you get a boyfriend or such, that you don’t just tell it right away

I’m the only one in school that suffer[s] from a mental illness. The others, they have ADHD or Asperger’s or depression… that’s all they’ve got. There’s no one with schizophrenia like me… And I feel like […] then, there’s not really anyone to talk to about… what it’s like. (Aasa, 15 years)

What emerges is a picture of interlocutors centralising different elements of the illness experience and making them the starting point of disclosure and shared understanding. Understanding becomes a negotiated concept through prioritising aspects of the illness experience (from having bad days to specific mental illness experiences). What differs is the centralisation of aspects. For some, having tough days is what is central, whereas others consider psychotic experiences central. This means that interlocutors have different needs when it comes to the background of experiences of those they disclose to. Some need a friend with a physical illness, whereas others specifically need people with experiences of psychosis to experience being understood − to know that they are not the only one.

The potential for creating the grounds for being understood and the experience of universality may be hampered by the age of the interlocutors, as their age peers will have had less lived experience of the crisis that Frede mentions, e.g., mental illness (which mostly debut later in life) and somatic illness. However, age is not a guarantee for being understood, as several interlocutors speak of adults whose reactions to disclosure the adolescents define as “not understanding”, e.g., as with Aasa, who mentions a “not understanding” teacher saying “get over that phase” and “just be normal again” in reaction to the disclosure of her mental illness.

## Discussion

To the best of our knowledge, our study is the first to explore the lived experiences of stigmatisation among adolescents with early onset psychosis. By focusing on not only the adolescents’ experiences of stigmatisation but also their processes of disclosure strategising, outcome evaluation and re-strategising, the study shows how even very young people suffering from severe mental illness are active agents navigating systems of stigma. This is in line with findings from studies of adults managing stigmatisation of psychosis, e.g., Jenkins and Carpenter-Song who conclude that the array of stigma management strategies reveal a social resourcefulness seldomly associated with people with mental illness (Jenkins & Carpenter-Song, 2008). Even though adolescents suffering from early onset psychosis are at a particularly fraught time in their lives, navigating both the developments of childhood and youth and the social disruption of psychotic illness, their narratives of stigma management show a potential for resilience. It is then up to families, therapists, teachers and others to support the development of this resilience.

The focus in our study was stigmatisation after diagnosis, but previous studies have found that stigma can have an impact even before diagnosis (Colizzi et al., 2020). Studying young people at risk for psychosis and bipolar disorder, Rusch et al. conclude that stigma stress predicted negative attitudes towards help-seeking (Rüsch et al., 2013). Research has also shown that stigma is a main barrier to help-seeking for young people (Gulliver et al., 2010) and has a disproportionate effect on help-seeking for adolescents compared with adults (Clement et al., 2015). Delays in help-seeking can in turn prolong the duration of untreated psychosis (DUP), which is associated with worse outcomes. As the average DUP in the TEA trial was approximately 30 months (median DUP 16 months; Pagsberg et al., 2017), our interlocutors may similarly have experienced a high risk of stigmatisation preceding diagnosis, which coupled with the DPM feedback loop, could impact their disclosure decision-making post-diagnosis.

Our study proposes two expansions of the DPM. Firstly, we argue that the model does not take the possible impact of outcomes of non-disclosure into account. Secondly, we highlight how external factors and considerations other than solely antecedent goals motivate and shape disclosure. One factor identified was the normativity of openness, which the study problematises within a social context where adolescents may face severe stigmatisation due to disclosure. As Jenkins and Carpenter-Song determine, concealment can be construed as a self-protective strategy in social contexts where attempts at therapeutic or political disclosure may not only fail, but can backfire (Jenkins & Carpenter-Song, 2008). Attempting to harness the possible benefits of disclosure, several researchers offer frameworks and decision aids to assist decisions on disclosure of mental health status (e.g., Henderson et al., 2012; Seeman, 2013). These frameworks, developed for adults, could be helpful for adolescents, but adolescents may have additional risks to consider regarding disclosure. Whereas Whitley and Campbell found that fear, much more so than experienced stigmatisation, was commonly experienced by their adult interlocutors recovering from severe mental illness (Whitley & Campbell, 2014), this is emphatically not the case for the interlocutors of our study. Considering the widespread experiences of stigmatisation described, as well as the possible social immaturity of peers, a central clinical implication of our study is therefore that care should be taken to ensure that adolescents suffering from psychosis are not being pressured into disclosure that they might not be ready for or feel is the right strategy.

An additional cause for caution regarding disclosure is the risk of illness identification. In *Reducing Self-Stigma by Coming Out Proud*, Corrigan et al. discuss how disclosure of mental illness can be beneficial in terms of reducing self-stigma but also detrimental, leading to illness identification, which can cause low self-esteem and pessimism (Corrigan et al., 2013). However, though the interlocutors do recount positive experiences of “being understood” by others with somewhat similar life experiences, they do not show signs of reframing psychosis as part of either their personal identity or group identity, as has been seen in recent years in the neurodiversity movement. The movement is a growing trend reframing, e.g., attention-deficit/hyperactivity disorder (ADHD) and autism spectrum disorder as aspects of identity, empowering people to embrace their differences (Armstrong, 2011; Kras, 2009). This reframing is not apparent in our analysis. The difference might be associated with the finding that the interlocutors often consider psychosis to be even more stigmatising than other mental illnesses (a finding consistent with studies of stigmatisation of psychosis in adults (Pescosolido et al., 2008)), and do not practice political disclosure of their early onset psychosis. It might be the case that the stigma of psychosis is so powerful that it is too socially perilous to reframe as part of one’s identity. Another reason might be that the interlocutors have either already attained remission or hope to do so in the future, and so consider their psychosis a temporary state, not comparable to permanent neurodiverse conditions. Unfortunately, recovery is not necessarily equivalent with the disappearance of risk of stigma, a predicament Jenkins and Carpenter-Song describes as “the paradox of stigma despite recovery” (Jenkins & Carpenter-Song, 2008).

The analysis highlights the centrality of the experience and impact of (not) being understood when sharing information about psychosis and comorbidities. This finding aligns with studies such as MacDonald et al. (2005) and Newton et al. (2007): shared experiences and being understood is a central theme of their analysis of interviews with, respectively, adolescents suffering from auditory hallucinations (Newton et al., 2007) and young adults recovering from first-episode psychosis (MacDonald et al., 2005). Though seldomly explored in-depth, this experiential element is alluded to by many researchers, e.g., Estroff et al., who in their introduction write “By definition and in essence, much of madness is invisible and unknowable to the ‘other’” (Estroff et al., 1991), and Meyer who argues that incomprehensibility breeds isolation and despair, and that re-establishing comprehensibility with one’s intimate others is crucial for recovery from psychosis (Myers, 2019). Our analysis develops the concept of being understood in two ways: first, by highlighting how the contrasting fear and experience of not being understood impacts the interlocutors’ choices about disclosure and, second, by showing how interlocutors are actively centralising aspects of their experience, thereby achieving the experience of being understood.

Our study was conducted through singular interviews. This spared the interlocuters longer-term involvement in the project, but also meant that they did not have the opportunity to revisit and amend their narratives. The study offers a rare insight into a group of adolescents whose experiences of stigma and management thereof are not often researched, but being a snapshot of their experiences does limit the scope of this study. As ‘(not) being understood’ emerged as a theme during analysis, rather than during data generation, it was not within the scope of this study to conduct further interviews to unfold the theme in more detail, to uncover more precisely what ‘being understood’ meant for and how it was defined by the interlocutors. Future research could delve into what constitutes ‘being understood’ to better support this process for the adolescents, for instance, through peer interventions.

Finally, the study provides the crucial clinical implication that it is not sufficient to consider the challenge of stigmatisation when trying to understand and guide adolescents suffering from early onset psychosis in terms of stigma management: the experience and fear of not being understood is not encapsulated within stigma definitions and is a central aspect to the experiences of adolescents with psychosis.

## Data Availability

The transcriptions of the interviews cannot be shared, as they contain identifying details about the participants.
